# The Placenta, How and When Delivered*Read before the Tri-State Medical Society of Alabama, Georgia and Tennessee.

**Published:** 1896-09

**Authors:** J. B. Murfree

**Affiliations:** Murfreesboro, Tenn.; President Tri-State Medical Society of Alabama, Georgia and Tennessee; Ex-President of Tennesse Medical Society; Professor of Surgery, Sewanee Medical College


### THE PLACENTA, HOW AND WHEN DELIVERED ?*

BY J. B. MURFREE, M. D., MURFREESBORO, TENN ,

President Tri-State Medical Society of Alabama, Georgia and Tennessee; Ex-President of Tennesse Medical Society; Professor of Surgery, Sewanee Medical College.

The expulsion of the secundines constitutes the third stage of labor. And though usually simple and naturally effected, yet it is of no small significance to the obstetrician, as not only the comfort, but sometimes even the safety of the woman depends on the safe delivery of the placenta.

The placenta consists essentially of two vascular membranes, one maternal, the other fœtal; so closely interlocked together that without any act of direct communication between the two vascular systems, interchanges of gases and nutritive and excretory material can and does take place between them.

It is a soft, spongy mass, constituting the principal connection between the ovum and the uterus, being destined for the hæmatosis, and perhaps also, for the nourishment of the child. It is described as a flattened body about three-fourths of an inch in thickness at the center, but tapering off towards the circumference, which does not often exceed two or three lines; in some cases it is very thin, and then it is very large in circumference and flattened.

Its figure and dimensions are exceedingly variable; thus the ordinary dimensions of the placenta vary from six to eight and a half inches. At times one diameter is larger than the other, hence the shape of the placenta may be circular, oval, or elongated.

*Read before the Tri-State Medical Society of Alabama, Georgia and Tennessee.

As a general rule but one placenta exists in a simple pregnancy; however, a double placenta for a single child has been found.

The uterine surface of the placenta is rough and uneven, slightly convex, and is divided into a number of lobes irregularly rounded off called cotyledons, held together by a thin texture (which is easily torn) and is covered by a thin layer of adhesive matter.

The fœtal side is covered by a layer of both the chorion and amnion, and has numerous ramifications of the vessels of the umbilical artery and veins, which converge to the center, to form the umbilical cord.

The placenta is regarded as originally being composed of two distinct parts or layers, a fœtal and maternal portion, which are so intimately woven together that it is impossible to separate them.

As soon as the formation of the placenta is begun the vessels of the uterus become enlarged, and the circulation of the blood through them quickened, and the diameter of the veins become so much increased as to constitute sinuses.

The uterine maternal side of the placenta is attached by means of the villi into the uterine walls, not by direct adhesion, but the insertion of the villi of the chorion into the crypts of the mucous membranes of the uterus. The arteries and veins are quite numerous throughout the placenta, and converge to the inner or fœtal surface to form the umbilical cord, which is most usually inserted into the center of this surface.

The placenta fulfills the function of oxygenation, nutrition and excretion to the fœtus.

The expulsion of the placenta and membranes completes the act of labor and is usually accomplished by nature; the last contractions of the uterus which expel the child are sometimes so violent as to drive out the placenta with the child; this, however, is rare. Most generally at the conclusion of the delivery of the child the placenta remains in the uterus, though partially detached, the contractions of that organ closing up the mouths of the open vessels, thereby preventing any undue hemorrhage. After labor, the uterus for an indefinite (usually short) period remains quiescent. The placenta has fulfilled its function, and is now a foreign body in the uterus, and this organ, responding to the irritation produced by it, soon begins to contract, and continues to contract until the placenta is forced into, and sometimes out of the vagina.

The time from the birth of the fœtus to the expulsion of the placenta, when left to the unaided powers of nature, varies greatly; from twenty minutes to an hour, sometimes an hour and a half or two hours. The proper management of the delivery of the placenta constitutes an important procedure in every case of labor. There is a difference of opinion among obstetricians upon this subject.

The child, being delivered, and its viability being fully manifested by its vociferous cries, should be separated from the mother by the severance of the cord and turned over to the nurse, after which the attention of the physician should be directed to the complete clearing of the uterine cavity, by the entire removal of all the secundines.

As before remarked, the placenta is now a foreign body and a source of irritation to the uterine surface. Having fulfilled its vital functions, it is now ready to be cast off as a useless encumbrance.

It has been said that at this stage of labor the woman is tired and worn out by the agonizing pains of travail in expelling the fœtus and needs rest, and should be let alone. Very true, but her condition is not such as to afford the needed and longed-for rest.

How long after the birth of the child should we wait for the expulsion of the placenta is an unsettled question.

My own practice is to be governed by the position of the placenta and the condition of the woman.

If the placenta has been expelled from the uterine cavity and is lying in the vagina, or if there is an undue hemorrhage, it ought to be removed at once.

There is nothing to be gained by delay in either of these conditions, for so long as the placenta lies in the vagina it only tends to obstruct the natural passage and favors the coagulation of blood in the os that may give rise to unnecessary and painful uterine contractions—perhaps a concealed hemorrhage. When there is profuse hemorrhage the retention of the placenta in the uterus interferes with its perfect contraction, the natural means of checking the hemorrhage.

To secure the prompt and safe delivery of the placenta, we should place one hand upon the hypogastrium and with gentle pressure follow up the uterus while the fœtus is being expelled, endeavoring to secure its prompt contraction afterward. After the delivery of the child the uterine contraction should be maintained, and to secure this end the hand should remain over the hypogastrium or substituted by that of the nurse. After the severance of the cord, gentle friction should be made over the uterus in order to stimulate its renewed contraction; at the same time gentle downward pressure is made, after the method of Crede. While thus manipulating the uterus we wait twenty minutes for the natural expulsion of the placenta. After this period the hand is introduced into the vagina, and if the placenta be felt it should be seized and removed; if, however, the placenta is still in the uterus, the cord is seized and gentle traction is made in the direction of the axis of the pelvis. Usually by gentle traction upon the cord the placenta is easily brought away *en masse* without tearing it, while the other hand upon the hypogastrium maintains a tonic contraction of the uterus. The method of Crede is the correct way of aiding the powers of nature in expelling the placenta; but it can be made more efficient by the help which the accoucheur can render by making gentle traction upon the cord.

After the birth of the child the placenta becomes a useless mass; having served its function it is no longer needed, but occupies the place of a foreign body and becomes a source of irritation, and should be delivered as soon as it can be done with comfort and safety to the mother.

There are two extremes into which we may fall.

One is a forcible removal of the placenta immediately upon the delivery of the child; the other waiting too long and relying entirely upon the unaided forces of nature to expel the placenta.

As soon as the child is born the placenta is usually detached from the uterus to a considerable extent, though not completely. And as a result of this detachment, there is a slight hemorrhage which usually is promptly arrested by the tonic contraction of the uterus, and in a short time more effectually by the closure of the mouths of the bleeding vessels by the formation of clots and thrombi. Hence to forcibly take away the placenta immediately after the birth of the child is to open again these vessels and expose the mother to unnecessary hemorrhage from want of uterine contraction, and the blocking up of the vessel by formation of thrombi.

On the contrary, to wait an indefinite time for the placenta to be expelled by the natural powers alone is to only prolong the discomfort of the woman, and to expose her to the danger of a too free hemorrhage. No case of labor is completed until the placenta, with its membranes, is delivered, and until this is accomplished there is no assured safety to the woman.

So long as the placenta remains in the uterus after the escape of the child, there is more or less danger; on more than one occasion I have seen a profuse hemorrhage while the placenta was in the uterus.

That a contracted uterus cannot bleed is an obstetrical aphorism, but a complete and permanent contraction of the uterus cannot occur and be maintained while its cavity is occupied by the placental mass. Therefore the retained placenta is a foreign body, and capable of doing harm, and ought to be removed as soon as it can be safely done, provided it is not expelled by the natural forces.

The time to deliver the placenta is after waiting a sufficient period to allow an opportunity for the uterine contractions to expel it, and if they fail to do so interference should be had and placenta delivered *per vim.*

How long we shall wait on the uterine contractions to expel the placenta is an unsettled question; authors differ. I believe the majority of practitioners wait too long; I do not believe any good is gained by waiting an hour or perhaps two, as is often done.

If the placenta is not expelled naturally in twenty minutes, the obstetrician should institute measures to remove it artificially, and free the woman of the encumbrance.

The manner of delivering it is, first, by making friction over the uterus with one hand over the hypogastrium, while at the same time with the other in the vagina, gentle traction is made upon the cord. With these efforts usually the placenta is readily brought away. Should they fail, all traction upon the cord for the time being must cease, while the kneading of the uterus must be continued.

After a few minutes of rest, traction should again be made upon the cord, and the uterus more energetically kneaded, at the same time the woman is encouraged to bear down, while gentle pressure is made in the direction of the axis of the pelvis.

Being foiled in this attempt the accoucheur should pass his hand up into the uterus and gently separate the placenta from its attachments and bring it away. Most generally the placenta presents with its edges in the os, which should be seized and brought away with a rotary motion.

Rarely, however, it presents with its center at the os, in which case traction upon the cord causes it to spread out like an umbrella and wedges it closer in the os. When the placenta presents with its center at the os, the proper treatment is to push back the central surface into the cavity, bring down one side of the placenta, and with a rotary motion remove it.

While objection has been urged against traction being made upon the cord in the delivery of the placenta, and unquestionably harm may be done when undue force is used, yet I insist that gentle traction can do no harm, while it materially aids the expulsive power of the uterus, and in the majority of the cases is a safe and successful procedure.

By gentle traction upon the cord we readily estimate the amount of resistance, and are able to some extent, at least, to determine the position of the placenta with the extent of its attachments.

Where there are extensive attachments no traction should be made upon the cord, but the hand should be introduced into the uterus and the placenta gently separated from it and brought away with a rotary motion, so as to wrap the membranes around it and secure their entire delivery. Undue force should not be used in making traction, or pulling upon the cord for fear of tearing it, which would be unfortunate, for the successful delivery of the placenta consists in removing it entirely.

